# Chiropractors in interprofessional practice settings: a narrative review exploring context, outcomes, barriers and facilitators

**DOI:** 10.1186/s12998-022-00461-1

**Published:** 2022-12-16

**Authors:** Corrie Myburgh, Solvej Teglhus, Kristian Engquist, Evgenios Vlachos

**Affiliations:** 1 Department of Sport Science and Clinical Biomechanics, University of Southern Denmark, Odense, Denmark; 2 Chiropractic Knowledge Hub, University of Southern Denmark, Odense, Denmark; 3 Department of Chiropractic, University of Johannesburg, Johannesburg, South Africa; 4 University Library of Southern Denmark, University of Southern Denmark, Odense, Denmark; 5 The Maersk Mc-Kinney Moller Institute, University of Southern Denmark, Odense, Denmark

**Keywords:** Interprofessional practice, Chiropractic, Review

## Abstract

To determine the added value of interprofessional interventions over existing mono-professional practice, elucidation of specific health care issues, service delivery contexts and benefits of combining multiple service provider is required. However, from existing literature, it is difficult to develop a sense of the evidence that supports interprofessional practice initiatives involving chiropractors. This review aims to describe and explore the contexts, outcomes, and barriers and facilitators relating to interprofessional practice involving chiropractors available in current literature. A search of Scopus, CINAHL, Cochrane, and Web of Science databases covering the literature from 2005 to October 2021 was conducted, after which a narrative review of identified peer-reviewed articles written in English was performed. We included data from seven studies, conducted across four distinct service delivery contexts. Eight interprofessional practice partners were identified, and eight factors appear to act as barriers and facilitators. Data suggests that incorporating chiropractors into community health and sports medicine interprofessional practice interventions is achievable and appears to impact collaborative practice positively. For older adults with low back pain, quality of life and care-related satisfaction are potential relevant outcomes for the evaluation of interprofessional practice interventions. There is currently very limited evidence from which to judge the value of interprofessional practice interventions, as available literature appears to focus mainly on interprofessional collaboration. Studies conducted specifically to evaluate interprofessional practice solutions and addressing specific health care issues or practice domains are urgently required.

## Background

**Fig. 1:**
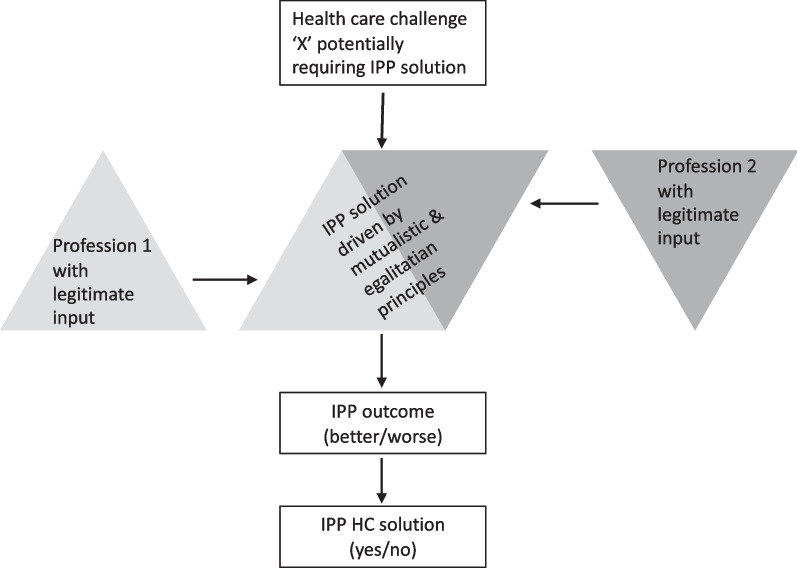
Development of a health care solution based on criteria and principles of interprofessional practice

In healthcare, interprofessional practice (IPP) exists under a specific set of circumstances. According to Parse [18], IPP occurs when two or more professional groups (with unique disciplinary knowledge) combine their services in order to provide a more optimized solution to a particular health care challenge. In its ideal form, IPP is guided by two key principles, these being mutualism and egalitarianism. With regards to the former, no single professional group claims the ability to provide the entire health care solution [1, 2]. And with regard to the latter, service delivery occurs in a collaborative, team-based context and as such "one profession does not preside over the others" [18], p. 5). Based on the guiding principles, the development of a particular health care solution (X) can be simply conceptualized as follows (see Fig. 1).

It is important not to view IPP as a panacea that adds value to every healthcare scenario. Simply introducing more health care partners is, in fact, just as likely to result in poorer service delivery outcomes [7, 11]. Rather, to extract maximum benefit from a multi-professional intervention, it is critical to clarify why the IPP approach is likely to provide a superior outcome, which professional competencies are required, and how the individual providers will function as a team [6, 8]. Moreover, and suffice is to say that the outcomes used to evaluate implementation and efficacy must bear out the hypothesized increased value [19, 20].

The chiropractic profession has recognized the importance of integrating its services with health care provider groups, who share a common interest in the management of musculoskeletal problems [9, 14]. As a result, investigations have begun to emerge focusing on how chiropractic services may feasibly be integrated into established health care settings [10, 13].

Based on current investigations, it is possible to develop an overview of how the efforts to embed chiropractors as IPP team members have been achieved, how they are perceived by other providers groups and indeed what factors tend to hinder and facilitate these efforts. However, it is perhaps more challenging, to develop a sense for which health care problems chiropractors might act effectively as IPP partners, and indeed whether these IPP solutions result in more favourable health care outcomes compared to existing practices.

With the aim of stimulating further discourse around this important topic, we were interested in exploring existing literature. We were specifically interested in describing the types of investigations that had been carried out, what factors facilitate and detract from service delivery initiatives and indeed whether evidence could be found for IPP practice solutions involving chiropractors providing better outcomes than existing (mono-professional) approaches.

## Identifying IPP initiatives involving chiropractors

### Search strategy

A search of Scopus, CINAHL, Cochrane, and Web of Science electronic databases was performed in free-text terms and in accordance with the PRISMA updated guidelines for conducting systematic reviews [17]. The search query was composed of three keywords: “interprofessional practice”, “healthcare” and “chiropractor” by random search and snowballing relevant terms and synonyms were added according to the defined keywords as described in specific for each of the chosen databases. The search was conducted in October 2021.

### Study selection and inclusion criteria

Two reviewers independently screened the articles in a three-step process: first the title, then the abstract, and finally the full text. In case of disagreement consensus was reached through discussion. An article was included if it described interprofessional practice including a chiropractor and outcome measurements relating to interprofessional service delivery were reported. In particular, articles were included if they had peer-reviewed scientific content in the form of journal articles, book chapters, and conference proceedings; were written in English; and, were published from 2005 till October 2021. The limitations on publication year were chosen after a random search was performed. The search showed no relevant research earlier than 2005. Furthermore, older studies may not be representative of the present trends, and therefore they were excluded.

For management of references and for identifying duplicates EndNote20.1 was used. Duplicates were identified by EndNote20.1 and then manually screened by both reviewers before removal.

### An example of study exclusion

We identified several studies that lay adjacent to our area of interest, offering important findings pertaining to the integration of chiropractors into multi-clinician practice settings [16, 25]. However, in order to highlight the status quo relating specifically to the domain of IPP interventions, we excluded studies where this focus could not be readily discerned. One such example, was the recent work of Whedon et al. [25] focusing on the primary spine care (PSC) clinician. We elected to exclude this work as part of our primary data sources, as it was not clear from either title or abstract that the work focused on the implementation of an IPP intervention. More specifically, the authors made use of the term multi-clinician primary care, which in our view, is not necessarily interchangeable with IPP.

In the same manner, articles on chiropractic services integration, interprofessional relations, and general chiropractic practice patterns were excluded.

## Scoping the evidence

### Studies describing interprofessional practice involving chiropractors

**Fig. 2:**
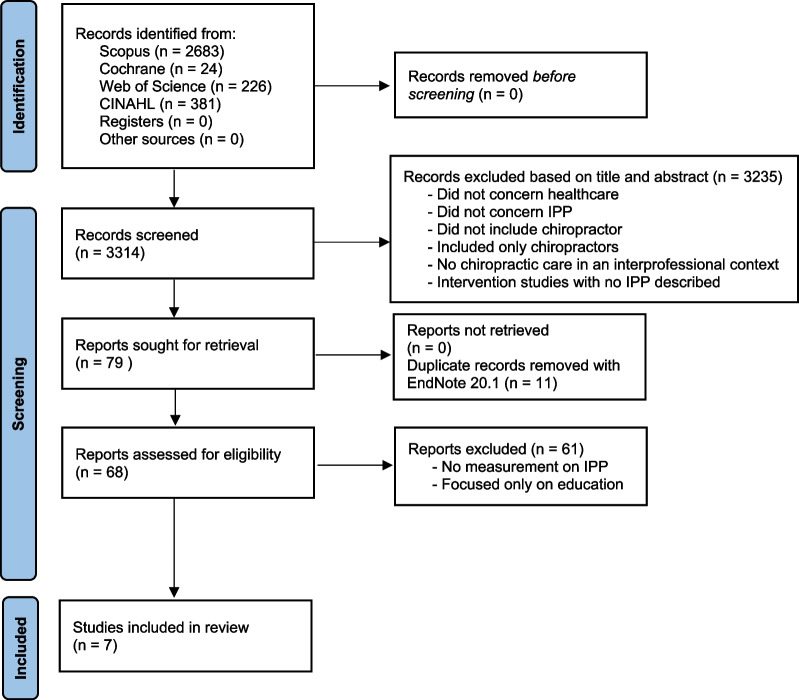
Schematic diagram of the identification of studies via databases based on the PRISMA 2020 flowchart [17]

The search resulted in 3314 articles: 2683 from Scopus, 24 from Cochrane, 226 from Web of Science, and 381 from CINAHL. After screening by title and abstract, 3235 articles were excluded due to the following reasons: did not include chiropractors or included only chiropractors; did not concern healthcare or did not involve healthcare settings; were not concerned with IPP; no chiropractic care in an interprofessional context; intervention studies with no IPP described (e.g., studies comparing IPP with non-IPP or the impact of adding chiropractic treatment compared with “normal care”); and, exploring IPP only in educational settings. In the remaining 79 articles, 11 duplicates were identified and removed, leaving 68 articles to be assessed for eligibility. Out of the 68 articles, 61 were excluded due to no measurements on IPP, or only measurements regarding interprofessional education, leaving 7 articles for inclusion. A schematic flowchart illustrates the search strategy in Fig. 2.

### Study characteristics

The studies (n = 7) were conducted in Canada (n = 3), the U.S.A. (n = 3), and Denmark (n = 1). As can be seen from Table 1, four studies investigated the integration of chiropractors or chiropractic services into interprofessional practice settings [9, 13, 22, 24]. One study explored aspects of interprofessional collaboration among complementary and integrative health providers (one of these being chiropractors) [21]. One study investigated aspects of interprofessional practice pertinent to chiropractic in elite football [12]. And finally, one study compared the effectiveness of guideline-based medical care, with the inclusion of chiropractic services either in parallel or as an integrated care package, on low back pain in older adults [10].

**Table 1:** Descriptive summary of studies relating to interprofessional practice involving chiropractors

Author and origin	Stated purpose	Context	Unit(s) of observation	Health care challenge	Design an Methods	IPP partners
[9] Canada	To investigate the effect of integrating chiropractic on the practice and attitudes of providers	Multidisciplinary healthcare teams at two community health centers	Service providers	None specified	*Design:* Mixed methods *Quantitative methods-*Provider Questionnaire regarding opinions, experiences with collaboration, and perceptions of chiropractic care *Qualitative methods*- Focus group interviews, individual interviews	MD Nurse practitioner Registered nurse
[24] Canada	To examine the integration of chiropractors into multi-disciplinary healthcare teams in the specialisation of sport medicine	High-performance sport work sites (e.g., major games, on tour with teams, at training centers)	Service providers	None specified	*Design:* Qualitative case study *Qualitative methods-* Individual interviews	MD PT Athletic therapist
[13] Canada	To evaluate if: Chiropractic could be integrated successfully within a hospital system The integration would reduce MSK pain and disability The level of satisfaction of patients and collaboration providers/administrators with the model of care	Hospital system of care- Department of Family and Community Medicine	Service providers and patients	Adults suffering from MSK pain and disability	*Design:* Prospective observational *Quantitative methods-* Self-reported pain, disability, general health, function, satisfaction *Qualitative methods-* Individual interviews	MD PT Administrators
[10] USA	To compare clinical outcomes of older adults receiving back pain treatment under three professional practice models that included primary medical care with or without chiropractic care	A family medicine residency and a chiropractic research center	Older adults (> 65)	LBP	*Design:* Pilot randomized controlled trial *Quantitative methods-* Primary outcomes: Self-reported LBP intensity & disability Secondary clinical outcomes: LBP bothersomeness, Fear Avoidance Beliefs, Timed Up and Go Test, perceived global improvement of LBP, overall health and quality of life and satisfaction with 6 domains of LBP care	MD
[22] USA	To evaluate the perceived feasibility of a patient-centered practice model for back pain, including facilitators for interprofessional collaboration between family MDs and chiropractors	A family medicine residency and a chiropractic research center located in Davenport, Iowa, USA	Service providers	LBP	*Design:* Qualitative evaluation *Qualitative methods*- individual interviews, chart abstractions, fieldnotes	MD
[21] USA	To investigate the aspects of interprofessional collaboration occurring in a sample of complementary and integrative health (CIH) providers	Private practice and community health centers	Service providers	None specified	*Design:* Qualitative health service case study *Qualitative methods*- Individual interviews	AOM practitioner Midwife Massage therapist Naturopath
[12]	Explore the role and perceived value of chiropractors to Danish elite football clubs. And additionally explore the barriers and facilitators to interprofessional practice involving chiropractors	Danish premier league (Superliga) clubs	Service coordinators	None specified	*Design:* Qualitative case study *Qualitative methods*- Individual interviews	None specified

*IPP* interprofessional practice, *LBP* low back pain, *MD* medical doctor, *PT* physiotherapist, *AOM* acupuncture and oriental medicine

Included studies were conducted across 4 distinct settings, these being: community health centers [9, 21], high-performance athletics [12, 24], a primary care hospital setting [13], and a training and research setting [10, 22].

Five investigations focused solely on service providers as units of observation [9, 12, 21, 22, 24]. The remaining two studies reported patient data or a combination of patient and provider data [10, 13].

Four investigations made use of qualitative designs [12, 21, 22, 24], 2 were mixed methods designs [9, 13] and one was a pilot randomized clinical trial [10].

Individual interviews were the most common method of observation, featuring in 5 investigations [12, 13, 21, 22, 24]. Clinical outcomes were captured in 2 studies [10, 13].

Eight distinct interprofessional practice partners were investigated, the most commonly involved being medical practitioners (6 studies) [9, 10, 12, 13, 22, 24], followed by physiotherapists (3 studies) [12, 13, 24].

### Barriers and facilitators and core findings relating to IPP

IPP-related findings, barriers and facilitators and are summarized in Table 2.

**Table 2:** Summary of findings, including barriers and facilitators to interprofessional practice

Reference	Key IPP-related findings	Facilitators	Barriers
[9]	Integrating chiropractic care into an established conventional medical setting, specifically a community health center, can be achieved over a relatively short period and with a high degree of comfort Integration had a positive impact on provider’s individual practices and changed opinions and views of healthcare practitioners towards chiropractic	The ‘right’ type of chiropractor that can integrate in an IPP setting IPE to ground the understanding of professional practice, roles and functions Collaborative practices such as team meetings and the clinic’s physical location Chiropractic care as a free service	Lingering concerns about ‘chiropractic’
[24]	The incorporation of chiropractic into the system of sport medicine professions derives from a clearly defined and commonly accepted understanding of the purpose of sport medicine Consumer demand (not professional acceptance) represents the main driver for the integration of chiropractors into high level athletics	A structural hierarchy that clarifies roles and responsibilities Clear understanding of professional boundaries and complementarity in each profession’s scope of practice Chiropractor’s accepting role limitations	Inability to work within a team framework Misaligned views regarding primary care status Misunderstandings about treatment provided by the chiropractor Clashes in philosophy of health stances
[13]	Even in the highly systematized settings of primary care hospital clinics, the addition of chiropractors, offers a useful addition to the management of MSK pain-related conditions	Credible champions leading the change towards IPP Thorough preparation of the IPP environment implementing IPP Clear support by senior level administration Chiropractor demonstrates a high level of profession expertise	Poor knowledge and of awareness of ‘the chiropractic service’ Perception of risk of chiropractic treatment Unstable funding model
[10]	Chiropractic services for back pain, offered either concurrently (dual care) or integrated (shared care) with standard medical care offers additional benefits beyond standard medical care alone with respect to patients global perceived improvement and satisfaction with care	Not reported	Not reported
[22]	Family medicine residents and Doctor of Chiropractic viewed collaborative care as a useful practice model for older adults with low back pain	IPE to establish, improve and maintain collaborative back pain care A team-based, patient-centered system of management practices An effective time scheduling and clinical records exchange platform Care facilities supportive of collaborative management	Care options unacceptable to patient due to high co-payment Inefficiencies in electronic journaling system Clinicians not participating in IPE are unlikely to refer patients to chiropractors
[21]	Complementary and integrative health providers collaborate both formally and informally with each other and other providers, and operate from a patient-centered perspective Providers overwhelmingly reported that interprofessional collaboration had a positive impact on patient care, professional satisfaction, and their practice	Collaborative practices such as interprofessional meetings Sharing supportive research for interventions with other providers and the target community Being present in a teaching hospital settings Creating clinical-experiential training sites allowing access to students from different disciplines Previous exposure to IPE Being in physical proximity of other providers Licensure, when required advised to consult with a medical doctor	The use of exclusive, discipline-specific language and nomenclature Clashes in professional opinions Poor of understanding about other disciplines Multiple electronic health records platforms Loss of income due to the extra time required to include collaborative practices Loss of income due to delays in treatment Licensure, when an obstacle to having hospital rights or limited the ability to bill third-party payers
[12]	Chiropractors are engaged in the role of a spine-related MSK health care expert, as part of a provider team. The rationale for adding a chiropractor is to broaden the shared pool of knowledge. However, when not utilized, the role of chiropractor as spinal health experts is challenged	Chiropractors shifting from external to in-house practitioners Perceived need for a (spinal) MSK expert Athlete demand for services provided by chiropractors	No clear niche for a Back Pain expert (Back pain perceived as self-remitting) Competition from existing provider groups Financial limitations to adding a chiropractor to the team

*IPP* interprofessional practice, *MSK* musculoskeletal, *IPE* interprofessional education

**Fig. 3:**
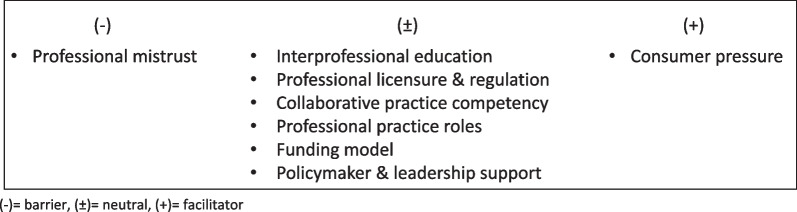
Factors associated with Interprofessional practice involving chiropractors. ( −) = barrier, ( ±) = neutral, ( +) = facilitator

Eight factors were identified as barriers and facilitators for IPP of which six of could act as either barrier or facilitator and were categorized as neutral. One factor, professional mistrust, was observed to act exclusively as a barrier, and was therefore categorized as negative. On the other hand, consumer pressure, was reported to act exclusively as a driver for IPP and was consequently categorized as purely positive in nature. The categorization of factors associated is illustrated in Fig. 3.

From available data, three core findings relating to chiropractors functioning in IPP contexts, were extracted and can be summarized as follows:

- IPP is achievable and impacts positively on collaborative practices in a community health care context;
- IPP potentially provides improved quality of life and care-related satisfaction outcomes for older adults with LBP, and;
- IPP in the sports medicine domain perceived as potentially desirable as long as the purpose is clear.

## Discussion

### Moving the state of the art forward

To our knowledge, this investigation is the first attempt at a meta-level evaluation of IPP interventions involving chiropractors. Based on our findings, and in an effort to integrate our findings with existing literature, we offer reflections under the following headings: investigatory focus; barriers and facilitators; IPP-related outcomes and developing the state of the art relating to IPP solutions.

### Investigatory focus

The overwhelming majority of articles we screened focused on developing an understanding of service provider interactions and the capturing user experiences. In our view, this focus is more in-tune with the concept of interprofessional collaboration (IPC), defined by Zwarenstein et al. [26] as ‘the process in which different professional groups work together to positively impact health care’*,* rather than that of IPP solutions. Indirect evidence for this argument can be seen in the commensurability of our findings with that of systematic review findings in the IPC context [26]. Similar to these data, we observed issues arising from conflicts in power dynamics, poor collaborative practice competency and a lack of clarity regarding roles and responsibilities as potential barriers to the inclusion of chiropractors.

In addition to the above, and seen from a methodological perspective, our data also reflected a focus on professional interactions, rather than the effectiveness of interventions. Specifically, the authors of our studies relied heavily on designs built around the acquisition of qualitative data [4, 5], which are best suited to research questions aimed at elucidating processes.

Thus, based on criteria of investigatory focus and research design, it’s likely that our data relates more closely to the context of interprofessional collaboration (IPC), rather than the effectiveness of IPP solutions. Specifically, our data largely addresses the processes (and issues) that influence adding a chiropractor to the provider team.

### Barriers and facilitators

Notwithstanding the apparent mismatch highlighted above, it would be prudent to remain cognizant of the barriers and facilitators identified in our investigation, as they are still likely to influence the implementation of IPP solutions. Among these, interprofessional education (IPE) and collaborative competency have been elucidated in some detail across available literature [19, 23].

With respect to IPE, the principle that people who learn together- work together, has gained significant traction amongst proponents of IPP. However, skepticism remains regarding the quality of evidence supporting the effect IPE exerts on patient outcomes. Based on the findings from their systematic review, Reeves et al. [19] concluded that it was still not possible to draw generalizable inferences about the effectiveness of IPE as a means of increasing IPP practice effectiveness. The authors suggested improvements in methodological rigor to document both successful IPE as well as IPP interventions, in order to strengthen a purported cause-effect relationship.

In relation to collaborative competency, a systematic review by Schot et al. [23], identified three key interactions occurring among professionals,these being ‘bridging gaps’, ‘negotiating overlaps’ and ‘creating professional practice spaces’. The authors concluded that effective IPC required and active effort and similar to Reeves et al. encouraged a research agenda that would focus on the nuances of successfully implementing IPC, paying particular attention to variations in IPP contexts, in order to improve patient outcomes.

Funding supportive of IPP was identified specifically as an issue pertaining to IPP solutions involving chiropractors. Although we categorized ‘funding’ as a neutral factor, it is clear from our primary investigation data that a poor understanding of reimbursement is likely to cripple interprofessional service provision endeavours [12, 21, 22]. Our findings are in tune with recent research conducted in a fee-for-service context and focusing on the introduction of chiropractors as primary spine care practitioners [3],). It would appear that, notwithstanding the offering being an efficient primary care service, poor reimbursement of the chiropractic clinician and high patient copayment, represent a significant structural barrier to service utilization [15, 16].

### IPP outcomes

In their systematic review focused on interprofessional practiced-based interventions, Zwarenstein et al. [26] argued that in order to develop the field, more investigations reporting mixed method data in single studies were required. More specifically, the authors called for cluster randomized studies reporting primary outcomes including measures such as patient-related quality of life and care-related satisfaction. Moreover, according to the authors, these outcomes should be supplemented by measures of IPC, and also supported by qualitative data elucidating the manner in which the IPP intervention influences collaboration.

As previously stated, our study included only one randomized intervention study, namely the pilot RCT conducted by Goertz et al. [10]. However, upon close scrutiny it appears that the investigation by Salsbury et al. [22], was conducted on the same patient population and at the same time. It is therefore possible to view these two investigations as the quantitative and qualitative elements of a mixed methods investigation. When viewed from this perspective, it seems plausible that investigators focused on the chiropractic profession have begun to conceptualize investigations in the manner suggested by Zwarenstein et al.

### Developing the discourse relating to IPP solutions

Given the nature of the evidence, and bearing in mind the heuristic framework presented (see in Fig. 1), we offer the following points as touchstones for future investigation.

Firstly, investigations relating to IPP involving chiropractors require greater conceptual clarity, so that the nature of the health care issue or the domain of practice being dealt with becomes clearer. Only with more studies addressing similar IPP focus areas, will it become possible to assess the level of evidence support intervention effectiveness. Secondly, and still in relation to conceptualization, greater clarity is required with regards to which other health care professionals chiropractors are partnered with and why this is likely to provide an advantageous outcome over a mono-professional solution. We would argue that establishing these two elements should be adopted as standard conceptualization practices and explicitly in IPP intervention studies.

Finally, the effective evaluation of IPP practice solutions require the reporting of outcomes that reflect both process and outcome. Thus, in order to determine whether any particular IPP practice solutions involving chiropractors provide a superior outcome to an existing (mono-professional) approach, outcomes are required that reflect the effectiveness of IPC as well as the IPP intervention itself.

### Limitations

The sample size of the included literature, the language selection, as well as the narrow distribution between countries could be identified as a limitation, thus only suggestions can be made to the global workforce of chiropractors. Regarding the selection of search terms, especially IPP and healthcare have many synonyms. To cover all possible synonyms a random search and snowballing were preformed, but undetected terms might have occurred. None of the included studies reported the use of specific validated instruments to measure IPP. However, they described the process of IPP in detail, had in-depth quality outcomes regarding the practitioners involved in IPP, explored the integration of chiropractic, and reported where, how, or if a chiropractor can contribute to a certain healthcare team.

## Conclusion

Very limited evidence from which to judge the value of IPP interventions involving chiropractors is currently available. Exploratory studies have outlined issues relating to feasibility and potential value of IPP initiatives across at least four domains of practice. However, only one study was identified with the specifically stated purpose of investigating an IPP practice intervention for a particular health care issue; this being low back pain in older patients. The discourse relating to IPP involving chiropractors appears to be at an early stage of development and further studies conducted specifically to evaluate IPP solutions for specific health care issues are urgently required.

## Data Availability

Not applicable.
